# Dr. Rich. Fawssett

**Published:** 1823-12-01

**Authors:** 


### OBITUARY.

Lately died at Horncastle, in Lincolnshire, Dr. Rich. Fawssett, very much regretted by an extensive circle of friends and patients.

